# Supplementary material for: A Likelihood Approach for Real-Time Calibration of Stochastic Compartmental Epidemic Models
Source: PLoS Comput Biol. 2017 Jan 17;13(1):e1005257. doi: 10.1371/journal.pcbi.1005257 (PMC5240920; doi:10.1371/journal.pcbi.1005257)
Supplement: S1 File — (TAR.GZ) [file pcbi.1005257.s014.tar.gz › HSPH_Online-SI-Revision/EnKF07/n10-extreme/n10-extreme_EnKF2_table8.pdf]

| | | | | | | | | | | | | | | | | | | | | | | | | | | | | | | |
|---|---|---|---|---|---|---|---|---|---|---|---|---|---|---|---|---|---|---|---|---|---|---|---|---|---|---|---|---|---|---|
| 1 | newly 3 weeks spec | 33. | 111.61 | {13., 472.} | 288.758 | 0.416575 | newly 3 weeks cumul | 135. | 941.82 | {140., 3242.} | 598.385 | 0.507883 | newly 5 weeks spec | 7. | 19.75 | {0., 79.} | 268.429 | 0.700182 | newly 5 weeks cumul | 159. | 1007.2 | {149., 3539.} | 536.327 | 0.474384 | newly 50 weeks cumul | 163. | 1023.74 | {152., 3628.} | 531.423 | 0.47389 |
| 2 | newly 3 weeks spec | 108. | 126.51 | {0., 127.} | 152.528 | 1.12144 | newly 3 weeks cumul | 418. | 905.93 | {214., 573.} | 165.294 | 0.266456 | newly 5 weeks spec | 44. | 41.06 | {0., 73.} | 133.227 | 1.58625 | newly 5 weeks cumul | 535. | 1017.4 | {238., 727.} | 154.673 | 0.302028 | newly 46 weeks cumul | 683. | 1096.26 | {242., 984.} | 138.738 | 0.34522 |
| 3 | newly 3 weeks spec | 12. | 260.79 | {0., 365.} | 2161.25 | 0.919953 | newly 3 weeks cumul | 49. | 584.61 | {23., 570.} | 1110.92 | 0.483179 | newly 5 weeks spec | 3. | 103.47 | {0., 458.} | 3466.33 | 1.21938 | newly 5 weeks cumul | 60. | 854.66 | {28., 2170.} | 1348.27 | 0.510658 | newly 52 weeks cumul | 67. | 1321.68 | {34., 7711.} | 1900.57 | 0.57327 |
| 4 | newly 3 weeks spec | 57. | 117.67 | {16., 176.} | 137.246 | 0.366812 | newly 3 weeks cumul | 214. | 1394.75 | {434., 2368.} | 558.715 | 0.621099 | newly 5 weeks spec | 34. | 22.18 | {0., 35.} | 65.7059 | 0.675767 | newly 5 weeks cumul | 292. | 1464.02 | {442., 2430.} | 408.425 | 0.530185 | newly 46 weeks cumul | 396. | 1492.41 | {442., 2434.} | 284.745 | 0.425935 |
| 5 | newly 3 weeks spec | 61. | 21.32 | {0., 44.} | 82. | 1.12916 | newly 3 weeks cumul | 281. | 846.78 | {115., 1908.} | 281.957 | 0.413013 | newly 5 weeks spec | 29. | 4.16 | {0., 15.} | 86.4828 | 1.55234 | newly 5 weeks cumul | 366. | 859.11 | {123., 1908.} | 229.09 | 0.460423 | newly 49 weeks cumul | 445. | 865.28 | {125., 1908.} | 200.297 | 0.51199 |
| 6 | newly 3 weeks spec | 52. | 63.56 | {5., 95.} | 133.885 | 0.622233 | newly 3 weeks cumul | 218. | 526.19 | {115., 918.} | 188.656 | 0.32631 | newly 5 weeks spec | 24. | 14.02 | {0., 21.} | 103.25 | 1.13016 | newly 5 weeks cumul | 283. | 568.84 | {117., 963.} | 164.155 | 0.362234 | newly 50 weeks cumul | 342. | 582.07 | {118., 966.} | 145.933 | 0.397426 |
| 7 | newly 3 weeks spec | 45. | 95.12 | {0., 180.} | 239.422 | 1.54006 | newly 3 weeks cumul | 222. | 1286.14 | {19., 2838.} | 530.288 | 0.676224 | newly 5 weeks spec | 26. | 98.1 | {0., 291.} | 405.154 | 1.4976 | newly 5 weeks cumul | 276. | 1482.35 | {24., 3350.} | 487.011 | 0.664914 | newly 51 weeks cumul | 350. | 1996.29 | {41., 8513.} | 517.711 | 0.660581 |
| 8 | newly 3 weeks spec | 74. | 353.98 | {0., 1725.} | 493.757 | 1.5172 | newly 3 weeks cumul | 355. | 1858.03 | {67., 5526.} | 458.786 | 0.531505 | newly 5 weeks spec | 40. | 136.19 | {0., 811.} | 360.425 | 1.44243 | newly 5 weeks cumul | 452. | 2220.39 | {90., 8414.} | 426.67 | 0.487406 | newly 48 weeks cumul | 577. | 2411.35 | {106., 10307.} | 357.315 | 0.444253 |
| 9 | newly 3 weeks spec | 75. | 113.86 | {10., 134.} | 134.053 | 0.486337 | newly 3 weeks cumul | 323. | 268.18 | {87., 403.} | 69.5418 | 0.373703 | newly 5 weeks spec | 31. | 203.54 | {1., 439.} | 629.419 | 0.725356 | newly 5 weeks cumul | 408. | 645.21 | {102., 811.} | 135.561 | 0.413415 | newly 48 weeks cumul | 485. | 1364.35 | {107., 7903.} | 252.101 | 0.489381 |
| 10 | newly 3 weeks spec | 97. | 155.27 | {0., 345.} | 204.031 | 1.415 | newly 3 weeks cumul | 429. | 1171.6 | {47., 4335.} | 256.918 | 0.55252 | newly 5 weeks spec | 65. | 35.99 | {0., 27.} | 130.108 | 2.03077 | newly 5 weeks cumul | 590. | 1291.17 | {50., 4766.} | 221.188 | 0.634055 | newly 48 weeks cumul | 747. | 1312.26 | {50., 4773.} | 189.724 | 0.695137 |
| 11 | newly 3 weeks spec | 96. | 69.87 | {0., 186.} | 115.948 | 1.11738 | newly 3 weeks cumul | 365. | 917.95 | {40., 2826.} | 259.247 | 0.631148 | newly 5 weeks spec | 43. | 20.49 | {0., 18.} | 109.837 | 1.35167 | newly 5 weeks cumul | 474. | 971.05 | {54., 3768.} | 220.973 | 0.662701 | newly 43 weeks cumul | 637. | 1022.51 | {57., 3770.} | 186.856 | 0.720891 |
| 12 | newly 3 weeks spec | 56. | 221.22 | {13., 500.} | 334. | 0.450049 | newly 3 weeks cumul | 258. | 1060.05 | {61., 3152.} | 342.624 | 0.455378 | newly 5 weeks spec | 30. | 92.31 | {4., 119.} | 271.9 | 0.518327 | newly 5 weeks cumul | 326. | 1295.66 | {79., 3653.} | 330.123 | 0.446419 | newly 48 weeks cumul | 370. | 1465.75 | {104., 3828.} | 328.046 | 0.442471 |
| 13 | newly 3 weeks spec | 46. | 104.71 | {31., 112.} | 140.761 | 0.247499 | newly 3 weeks cumul | 235. | 454.51 | {242., 507.} | 97.7745 | 0.198095 | newly 5 weeks spec | 40. | 59.71 | {5., 87.} | 111.375 | 0.37689 | newly 5 weeks cumul | 321. | 593.75 | {294., 621.} | 90.6137 | 0.164839 | newly 44 weeks cumul | 406. | 903.69 | {315., 1616.} | 131.126 | 0.205495 |
| 14 | newly 3 weeks spec | 50. | 56.06 | {0., 127.} | 99.2 | 0.831765 | newly 3 weeks cumul | 229. | 2595.13 | {194., 11805.} | 1044.69 | 0.624249 | newly 5 weeks spec | 25. | 27.99 | {0., 72.} | 116.92 | 1.36225 | newly 5 weeks cumul | 291. | 2662. | {264., 11805.} | 826.495 | 0.587703 | newly 49 weeks cumul | 352. | 2740.75 | {316., 11805.} | 691.366 | 0.56907 |
| 15 | newly 3 weeks spec | 41. | 51.3 | {0., 82.} | 115.707 | 0.879207 | newly 3 weeks cumul | 175. | 879.34 | {90., 3195.} | 419.931 | 0.50757 | newly 5 weeks spec | 20. | 13.53 | {0., 26.} | 110.95 | 1.36942 | newly 5 weeks cumul | 229. | 915.82 | {98., 3276.} | 317.59 | 0.427755 | newly 46 weeks cumul | 253. | 950.74 | {159., 3277.} | 290.577 | 0.399874 |
| 16 | newly 3 weeks spec | 68. | 47.94 | {0., 117.} | 65.7353 | 0.890618 | newly 3 weeks cumul | 284. | 1691.29 | {159., 9064.} | 510.496 | 0.396701 | newly 5 weeks spec | 35. | 21.15 | {0., 58.} | 73.3429 | 1.25438 | newly 5 weeks cumul | 368. | 1743.98 | {245., 9066.} | 390.56 | 0.351085 | newly 49 weeks cumul | 425. | 1806.13 | {313., 9066.} | 338.624 | 0.328769 |
| 17 | newly 3 weeks spec | 58. | 123.36 | {0., 143.} | 243.655 | 1.7374 | newly 3 weeks cumul | 228. | 1916.68 | {60., 6950.} | 770.789 | 0.691477 | newly 5 weeks spec | 27. | 106.98 | {0., 86.} | 423.111 | 1.61874 | newly 5 weeks cumul | 296. | 2143.21 | {75., 7637.} | 652.551 | 0.646364 | newly 51 weeks cumul | 355. | 2568.97 | {100., 10390.} | 647.372 | 0.636279 |
| 18 | newly 3 weeks spec | 58. | 27.64 | {0., 68.} | 61.3448 | 0.841733 | newly 3 weeks cumul | 249. | 1216.42 | {137., 3257.} | 422.41 | 0.318662 | newly 5 weeks spec | 33. | 10.39 | {0., 39.} | 78.697 | 1.50388 | newly 5 weeks cumul | 331. | 1243.17 | {145., 3257.} | 324.976 | 0.345606 | newly 49 weeks cumul | 406. | 1264.37 | {145., 3257.} | 272.549 | 0.378097 |
| 19 | newly 3 weeks spec | 19. | 25.52 | {0., 41.} | 163.684 | 1.40966 | newly 3 weeks cumul | 129. | 2402.14 | {55., 10628.} | 1791.07 | 0.867225 | newly 5 weeks spec | 10. | 19.38 | {0., 20.} | 225.4 | 1.38505 | newly 5 weeks cumul | 154. | 2443.56 | {77., 10628.} | 1511.03 | 0.810207 | newly 51 weeks cumul | 172. | 2519.38 | {99., 10628.} | 1380.13 | 0.77276 |
| 20 | newly 3 weeks spec | 82. | 284.67 | {5., 1312.} | 327.183 | 0.643136 | newly 3 weeks cumul | 349. | 804.35 | {32., 2644.} | 208.387 | 0.570251 | newly 5 weeks spec | 51. | 173.61 | {1., 620.} | 328.686 | 0.778765 | newly 5 weeks cumul | 464. | 1217.17 | {38., 5774.} | 240.394 | 0.594137 | newly 48 weeks cumul | 599. | 1575.94 | {38., 8531.} | 235.232 | 0.610645 |
| 21 | newly 3 weeks spec | 71. | 169.65 | {0., 354.} | 234.634 | 1.39487 | newly 3 weeks cumul | 319. | 2130.21 | {181., 8093.} | 589.063 | 0.561033 | newly 5 weeks spec | 24. | 96.92 | {0., 202.} | 392.25 | 1.30419 | newly 5 weeks cumul | 400. | 2353.65 | {245., 8093.} | 509.653 | 0.547216 | newly 45 weeks cumul | 509. | 2862.14 | {273., 8425.} | 482.428 | 0.562946 |
| 22 | newly 3 weeks spec | 15. | 247.22 | {5., 928.} | 1576.8 | 0.834287 | newly 3 weeks cumul | 86. | 2064.33 | {135., 8112.} | 2302.41 | 0.765613 | newly 5 weeks spec | 10. | 100.88 | {0., 159.} | 973. | 1.02835 | newly 5 weeks cumul | 114. | 2303.48 | {152., 9121.} | 1923.47 | 0.736039 | newly 52 weeks cumul | 122. | 2687.88 | {152., 9245.} | 2106.62 | 0.796347 |
| 23 | newly 3 weeks spec | 49. | 46.7 | {0., 68.} | 120.49 | 1.0169 | newly 3 weeks cumul | 175. | 834.43 | {132., 949.} | 401.949 | 0.307457 | newly 5 weeks spec | 26. | 11.54 | {0., 27.} | 95.6923 | 1.39101 | newly 5 weeks cumul | 236. | 867.44 | {140., 994.} | 313.008 | 0.334538 | newly 47 weeks cumul | 286. | 905.58 | {142., 1653.} | 277.091 | 0.387016 |
| 24 | newly 3 weeks spec | 62. | 91.87 | {0., 337.} | 165.887 | 1.67963 | newly 3 weeks cumul | 297. | 1659.84 | {168., 5313.} | 479.609 | 0.503334 | newly 5 weeks spec | 38. | 82.02 | {0., 180.} | 234.579 | 1.60167 | newly 5 weeks cumul | 391. | 1828.24 | {218., 8135.} | 391.857 | 0.487726 | newly 45 weeks cumul | 543. | 2344.66 | {249., 8424.} | 358.744 | 0.499871 |
| 25 | newly 3 weeks spec | 17. | 119.81 | {16., 105.} | 611.235 | 0.448771 | newly 3 weeks cumul | 104. | 342.02 | {141., 388.} | 228.865 | 0.329388 | newly 5 weeks spec | 9. | 82.41 | {2., 59.} | 856.333 | 0.586549 | newly 5 weeks cumul | 127. | 532.99 | {151., 527.} | 319.709 | 0.344909 | newly 48 weeks cumul | 139. | 698.08 | {158., 703.} | 402.403 | 0.387784 |
| 26 | newly 3 weeks spec | 8. | 79.01 | {0., 62.} | 1038.63 | 1.66192 | newly 3 weeks cumul | 30. | 978.13 | {53., 1542.} | 3169.17 | 1.17473 | newly 5 weeks spec | 1. | 102.87 | {0., 39.} | 10333. | 1.18429 | newly 5 weeks cumul | 36. | 1178.85 | {66., 4099.} | 3183.14 | 1.14647 | newly 51 weeks cumul | 40. | 1592.46 | {101., 8175.} | 3889.1 | 1.1583 |
| 27 | newly 3 weeks spec | 65. | 37.31 | {0., 87.} | 65.8 | 0.945044 | newly 3 weeks cumul | 303. | 895.77 | {62., 675.} | 234.591 | 0.334656 | newly 5 weeks spec | 45. | 25.52 | {0., 50.} | 85.5111 | 1.27124 | newly 5 weeks cumul | 394. | 949.07 | {64., 707.} | 186.855 | 0.329075 | newly 49 weeks cumul | 475. | 1051.52 | {98., 717.} | 173.246 | 0.342072 |
| 28 | newly 3 weeks spec | 25. | 268.02 | {0., 823.} | 1085.04 | 1.29249 | newly 3 weeks cumul | 120. | 1056.88 | {27., 4973.} | 807.067 | 0.577629 | newly 5 weeks spec | 9. | 57.8 | {0., 259.} | 688.222 | 1.56693 | newly 5 weeks cumul | 146. | 1233.43 | {27., 5872.} | 772.158 | 0.548813 | newly 52 weeks cumul | 160. | 1401.54 | {27., 7335.} | 804.725 | 0.555084 |
| 29 | newly 3 weeks spec | 36. | 116.61 | {0., 218.} | 304.139 | 0.755331 | newly 3 weeks cumul | 170. | 1007.71 | {302., 1230.} | 493.841 | 0.516487 | newly 5 weeks spec | 12. | 34.92 | {0., 60.} | 310.833 | 1.22346 | newly 5 weeks cumul | 201. | 1108.79 | {311., 1826.} | 452.572 | 0.473267 | newly 47 weeks cumul | 219. | 1161.47 | {311., 2501.} | 430.963 | 0.451203 |
| 30 | newly 3 weeks spec | 50. | 141.72 | {0., 193.} | 288.36 | 1.41018 | newly 3 weeks cumul | 199. | 1273.68 | {51., 2680.} | 564.211 | 0.682259 | newly 5 weeks spec | 23. | 93.34 | {0., 169.} | 415.913 | 1.54724 | newly 5 weeks cumul | 254. | 1482.07 | {61., 3254.} | 507.374 | 0.655046 | newly 47 weeks cumul | 304. | 1913.22 | {88., 4212.} | 550.566 | 0.666316 |
| 31 | newly 3 weeks spec | 53. | 55.26 | {4., 44.} | 121.66 | 0.581091 | newly 3 weeks cumul | 252. | 438. | {94., 273.} | 153.825 | 0.348428 | newly 5 weeks spec | 36. | 10.19 | {0., 15.} | 91.5833 | 1.26355 | newly 5 weeks cumul | 334. | 471.55 | {97., 335.} | 137.189 | 0.410585 | newly 50 weeks cumul | 377. | 479.48 | {97., 343.} | 130.531 | 0.443768 |
| 32 | newly 3 weeks spec | 76. | 90.67 | {11., 328.} | 118.776 | 0.611824 | newly 3 weeks cumul | 281. | 391.09 | {68., 1316.} | 125.107 | 0.528194 | newly 5 weeks spec | 36. | 52.32 | {1., 167.} | 146.278 | 0.729642 | newly 5 weeks cumul | 374. | 510.09 | {89., 1653.} | 125.644 | 0.553904 | newly 42 weeks cumul | 533. | 764.03 | {144., 2441.} | 139.612 | 0.617043 |
| 33 | newly 3 weeks spec | 8. | 62.02 | {8., 121.} | 683.75 | 0.609721 | newly 3 weeks cumul | 52. | 337.12 | {81., 884.} | 553.308 | 0.575001 | newly 5 weeks spec | 2. | 33.95 | {0., 53.} | 1627.5 | 0.85528 | newly 5 weeks cumul | 59. | 415.73 | {111., 941.} | 608.424 | 0.601189 | newly 50 weeks cumul | 62. | 609.77 | {137., 1272.} | 887.048 | 0.703461 |
| 34 | newly 3 weeks spec | 8. | 226.91 | {10., 838.} | 2740.13 | 0.795828 | newly 3 weeks cumul | 61. | 1144.91 | {177., 2694.} | 1776.9 | 0.828575 | newly 5 weeks spec | 7. | 149.87 | {0., 389.} | 2107.57 | 0.787087 | newly 5 weeks cumul | 76. | 1495.88 | {178., 5839.} | 1868.26 | 0.798183 | newly 52 weeks cumul | 80. | 1686.1 | {179., 7737.} | 2007.63 | 0.796031 |
| 35 | newly 3 weeks spec | 95. | 63.89 | {0., 98.} | 112.811 | 1.37684 | newly 3 weeks cumul | 347. | 802.3 | {225., 532.} | 174.646 | 0.260245 | newly 5 weeks spec | 52. | 12.96 | {0., 27.} | 97. | 1.77597 | newly 5 weeks cumul | 464. | 842.66 | {227., 617.} | 146.845 | 0.305193 | newly 47 weeks cumul | 569. | 858.54 | {228., 710.} | 132.359 | 0.354918 |
| 36 | newly 3 weeks spec | 110. | 232.13 | {0., 858.} | 199.409 | 1.31869 | newly 3 weeks cumul | 389. | 2534.8 | {187., 8382.} | 566.73 | 0.587282 | newly 5 weeks spec | 47. | 128.63 | {0., 324.} | 263.681 | 1.2183 | newly 5 weeks cumul | 515. | 2836.44 | {243., 8551.} | 466.206 | 0.544649 | newly 45 weeks cumul | 717. | 3219.26 | {356., 8682.} | 364.371 | 0.479506 |
| 37 | newly 3 weeks spec | 60. | 330.19 | {0., 1663.} | 556.517 | 1.2785 | newly 3 weeks cumul | 245. | 1730.53 | {173., 7819.} | 625.147 | 0.498862 | newly 5 weeks spec | 22. | 134.89 | {0., 251.} | 640.5 | 1.57617 | newly 5 weeks cumul | 316. | 2086.74 | {176., 8816.} | 589.665 | 0.508589 | newly 48 weeks cumul | 370. | 2208.04 | {176., 8880.} | 534.319 | 0.507936 |
| 38 | newly 3 weeks spec | 10. | 61.67 | {0., 39.} | 655.1 | 1.53373 | newly 3 weeks cumul | 32. | 875.15 | {53., 871.} | 2638.84 | 1.00754 | newly 5 weeks spec | 1. | 63.95 | {0., 22.} | 6437. | 1.10657 | newly 5 weeks cumul | 36. | 1003.28 | {66., 2026.} | 2690.44 | 1.00548 | newly 51 weeks cumul | 37. | 1331.44 | {72., 5486.} | 3501.89 | 1.04779 |
| 39 | newly 3 weeks spec | 14. | 57.26 | {0., 161.} | 411.714 | 1.28534 | newly 3 weeks cumul | 85. | 1579.05 | {41., 5856.} | 1771.96 | 0.941536 | newly 5 weeks spec | 6. | 46.27 | {0., 111.} | 787.833 | 1.39859 | newly 5 weeks cumul | 101. | 1674.8 | {67., 5856.} | 1570.55 | 0.919436 | newly 51 weeks cumul | 112. | 2076.18 | {202., 6480.} | 1761.05 | 0.960402 |
| 40 | newly 3 weeks spec | 73. | 67.4 | {6., 55.} | 120.164 | 0.659312 | newly 3 weeks cumul | 282. | 182.71 | {66., 228.} | 74.5496 | 0.473437 | newly 5 weeks spec | 30. | 85.23 | {0., 32.} | 308.833 | 0.8596 | newly 5 weeks cumul | 357. | 353.67 | {74., 291.} | 111.678 | 0.519183 | newly 48 weeks cumul | 439. | 535.07 | {75., 454.} | 137.806 | 0.565302 |
| 41 | newly 3 weeks spec | 66. | 189.02 | {12., 425.} | 225. | 0.484378 | newly 3 weeks cumul | 240. | 1788.94 | {102., 5381.} | 668.225 | 0.632994 | newly 5 weeks spec | 31. | 75.96 | {0., 137.} | 228.645 | 0.776483 | newly 5 weeks cumul | 309. | 1970.23 | {136., 6092.} | 561.395 | 0.611134 | newly 47 weeks cumul | 381. | 2277.51 | {202., 6759.} | 518.018 | 0.606813 |
| 42 | newly 3 weeks spec | 69. | 80.26 | {18., 155.} | 81.8841 | 0.30461 | newly 3 weeks cumul | 344. | 345.27 | {154., 630.} | 55.9157 | 0.241877 | newly 5 weeks spec | 67. | 49.22 | {5., 87.} | 97.1642 | 0.651539 | newly 5 weeks cumul | 474. | 456.39 | {199., 839.} | 65.8966 | 0.285957 | newly 42 weeks cumul | 655. | 648.43 | {215., 1173.} | 81.9038 | 0.345786 |
| 43 | newly 3 weeks spec | 15. | 317.36 | {4., 917.} | 2044.53 | 0.942442 | newly 3 weeks cumul | 71. | 1694.77 | {124., 3741.} | 2288.8 | 0.903796 | newly 5 weeks spec | 6. | 230.69 | {0., 666.} | 3797.5 | 1.21474 | newly 5 weeks cumul | 88. | 2202.46 | {133., 6745.} | 2405.36 | 0.931345 | newly 52 weeks cumul | 93. | 2689.69 | {133., 7824.} | 2794.96 | 0.983119 |
| 44 | newly 3 weeks spec | 89. | 69.86 | {1., 160.} | 75.8876 | 0.788411 | newly 3 weeks cumul | 399. | 1603.28 | {143., 5879.} | 326.717 | 0.451155 | newly 5 weeks spec | 51. | 22.93 | {0., 70.} | 77.2745 | 1.40128 | newly 5 weeks cumul | 518. | 1663.86 | {163., 5880.} | 248.506 | 0.406624 | newly 43 weeks cumul | 705. | 1716.42 | {178., 5880.} | 177.399 | 0.362172 |
| 45 | newly 3 weeks spec | 84. | 186.8 | {0., 756.} | 240.429 | 1.36179 | newly 3 weeks cumul | 323. | 1500.8 | {297., 5503.} | 370.669 | 0.321432 | newly 5 weeks spec | 39. | 62.07 | {0., 142.} | 199.051 | 1.5404 | newly 5 weeks cumul | 423. | 1667.83 | {301., 7007.} | 315.52 | 0.318171 | newly 47 weeks cumul | 539. | 1765.3 | {301., 7798.} | 267.688 | 0.342467 |
| 46 | newly 3 weeks spec | 113. | 226.23 | {0., 483.} | 191.088 | 1.45947 | newly 3 weeks cumul | 424. | 1911.96 | {288., 5630.} | 360.495 | 0.457739 | newly 5 weeks spec | 54. | 151.46 | {0., 434.} | 270.519 | 1.37218 | newly 5 weeks cumul | 553. | 2246.94 | {321., 6778.} | 316.919 | 0.434775 | newly 45 weeks cumul | 782. | 2737.44 | {407., 8928.} | 264.09 | 0.393211 |
| 47 | newly 3 weeks spec | 79. | 186.56 | {0., 330.} | 235.215 | 1.36768 | newly 3 weeks cumul | 324. | 1485.67 | {146., 2738.} | 377.096 | 0.534233 | newly 5 weeks spec | 43. | 123.52 | {0., 353.} | 288.651 | 1.5205 | newly 5 weeks cumul | 419. | 1757.81 | {235., 3492.} | 337.11 | 0.509473 | newly 47 weeks cumul | 531. | 2392.28 | {390., 7770.} | 365.232 | 0.532943 |
| 48 | newly 3 weeks spec | 58. | 67.5 | {6., 106.} | 122.138 | 0.5571 | newly 3 weeks cumul | 234. | 530.64 | {117., 1113.} | 173.786 | 0.337309 | newly 5 weeks spec | 22. | 14.01 | {0., 20.} | 103.136 | 1.0032 | newly 5 weeks cumul | 292. | 575.28 | {121., 1152.} | 153.904 | 0.350122 | newly 50 weeks cumul | 344. | 588.9 | {121., 1161.} | 138.855 | 0.374101 |
| 49 | newly 3 weeks spec | 22. | 55.93 | {0., 124.} | 281.136 | 1.37783 | newly 3 weeks cumul | 126. | 1464.99 | {49., 3739.} | 1089.98 | 0.795683 | newly 5 weeks spec | 10. | 50.1 | {0., 69.} | 538.4 | 1.47368 | newly 5 weeks cumul | 151. | 1568.85 | {63., 4081.} | 965.53 | 0.762459 | newly 51 weeks cumul | 165. | 1819.99 | {79., 8251.} | 1026.48 | 0.755132 |
| 50 | newly 3 weeks spec | 52. | 33.11 | {2., 70.} | 59.5577 | 0.657194 | newly 3 weeks cumul | 223. | 882.96 | {121., 2479.} | 310.529 | 0.495206 | newly 5 weeks spec | 29. | 3.18 | {0., 6.} | 95.931 | 1.85923 | newly 5 weeks cumul | 274. | 894.58 | {122., 2487.} | 243.38 | 0.435921 | newly 49 weeks cumul | 323. | 904.38 | {122., 2488.} | 198.57 | 0.388159 |
